# A Fair Contention Access Scheme for Low-Priority Traffic in Wireless Body Area Networks

**DOI:** 10.3390/s17091931

**Published:** 2017-08-23

**Authors:** Shagufta Henna, Muhammad Sajeel, Faisal Bashir, Muhammad Asfand-e-yar, Muhammad Tauqir

**Affiliations:** Department of Computer Science, Bahria University, Islamabad 44000, Pakistan; sajeel@isb.phoenix.com.pk (M.S.); hodcsib@Bahria.edu.pk (F.B.); m.asfandyar@bui.edu.pk (M.A.); tauqir130@gmail.com (M.T.)

**Keywords:** WBAN, MAC superframe structure, low-priority traffic, priority-based MAC, PA-MAC

## Abstract

Recently, wireless body area networks (WBANs) have attracted significant consideration in ubiquitous healthcare. A number of medium access control (MAC) protocols, primarily derived from the superframe structure of the IEEE 802.15.4, have been proposed in literature. These MAC protocols aim to provide quality of service (QoS) by prioritizing different traffic types in WBANs. A contention access period (CAP)with high contention in priority-based MAC protocols can result in higher number of collisions and retransmissions. During CAP, traffic classes with higher priority are dominant over low-priority traffic; this has led to starvation of low-priority traffic, thus adversely affecting WBAN throughput, delay, and energy consumption. Hence, this paper proposes a traffic-adaptive priority-based superframe structure that is able to reduce contention in the CAP period, and provides a fair chance for low-priority traffic. Simulation results in ns-3 demonstrate that the proposed MAC protocol, called traffic- adaptive priority-based MAC (TAP-MAC), achieves low energy consumption, high throughput, and low latency compared to the IEEE 802.15.4 standard, and the most recent priority-based MAC protocol, called priority-based MAC protocol (PA-MAC).

## 1. Introduction

Recently, wireless networks have rapidly advanced with respect to autonomous communication with diverse device connectivity. These networks target high data rates with a requirement of longer distances. The expansion of the IEEE 802.11 series has caused an increase in data rates, along with an increase in the communication range. Bluetooth, which is based on the IEEE 802.15.1 standard, is for short-range applications, and is designed for small devices. The complexity of bluetooth devices incurs a higher cost, and makes it unsuitable for applications demanding less power consumption and lower costs [1]. One of the specifications of IEEE 802.15.3, known as the ultra wideband (UWB), also aims to achieve higher data rates, and therefore is not suitable for low data rate-demanding applications [1]. Low rate wireless personal area networks (LR-WPAN ) have the requirements of short range, low power, and low data rate communication. In order to meet the growing demands of LR-WPAN applications, IEEE 802.15.4 has been introduced.

Wireless sensor networks (WSNs), have recently emerged with unique applications in physical security, healthcare, and commerce. Wireless body area networks (WBANs), are wireless networks of wearable or implantable computing devices. A WBAN consists of low cost and lightweight sensors like an electrocardiogram (ECG), a breathing sensor, a blood pressure sensor, or a glucose sensor etc. In early 1990s, the idea of communications within the human body gained popularity, and led to the birth of short-range communication using IEEE 802.15.4 wireless personal area networks (WPANs), to implement communications around the human body. A WBAN may use different WPAN tools as gateways to connect with other wireless technologies for longer distances, e.g., Internet. In this way, medical professionals can monitor the patient’s health related data online regardless of the patient’s location, with 24-h health-care support [2]. Recently, there has been a trend for enabling efficient WBAN-specific signal-processing applications to develop rapid prototyping of WBAN applications [3].

The IEEE 802.15.4 standard defines physical layer and medium access control (MAC) layer specifications. The physical layer mainly provides physical layer services, including energy detection, clear channel assessment, channel selection, and link quality indicatios. It operates in unlicensed frequency bands including 868.0–868.6 MHz, 902–928 MHz, and 2400–2483.5 MHz, with a limited range of communication channels. The MAC layer of IEEE 802.15.4 uses the services of the physical layer for data transmission. In addition to data transmission, the MAC layer also provides interface management services including association, disassociation, frame validation, and synchronization by using special beacons.

Traffic generation by sensors in a WBAN can be classified as normal, on-demand, and as emergency traffic. Normal traffic consists of routine data collected by sensor nodes for periodic monitoring of health conditions, e.g., temperature, glucose, and blood pressure [4]. On-demand traffic is explicitly requested by a coordinator to acquire specific information, e.g., ECGs, and electromyograms (EMGs), etc. [5]. On the other hand, emergency traffic is irregular traffic, which may occur anytime in response to some critical and unpredictable medical events, e.g., a drop in oxygen saturation level, and cardiac arrest etc. [2]. The unpredictable nature of emergency events and the strict latency requirements to report an event may make these situations difficult to handle.

The IEEE 802.15.6 standard [6], complements the physical(PHY) and MAC layers of IEEE 802.15.4 to specifically support medical applications, including emergency situations. IEEE 802.15.6 seems a better candidate for inexpensive medical applications with strict reliability requirements, however, its specification details are not completely disclosed. Due to lack of maturity of IEEE 802.15.6, a better approach is to design a WBAN system based on the IEEE 802.15.4 standard, which has already been applied to many fields including healthcare. To some extent, the use of IEEE 802.15.4 meets the requirements of WBAN to achieve a low data rate, low latency, and short range. Its use has been successfully reported to monitor ECGs, and it has been well applied to other WBAN platforms [7]. However, its use has demonstrated significant limitations for its practical implementation when applied for special purposes, where it fails to fulfill some of the inherent requirements of WBANs. IEEE 802.15.4 provides no built-in mechanism for traffic prioritization and service differentiation, and therefore, treats all traffic categories including emergency traffic the same way. The inability of IEEE 802.15.4 to handle unpredictable traffic situations may result in higher latency, which may worsen a critical situation.

In order to provide traffic prioritization, several enhancements to the IEEE 802.15.4 standard have been proposed in literature. In the most recent work, a priority-based MAC protocol, PA-MAC [8] is proposed to prioritize the traffic according to different traffic classes. In PA-MAC, the contention access period (CAP) is divided into four sub-phases according to the traffic priority. CAP period is highly contended by all traffic priorities, resulting in higher number of collisions and re-transmissions. Further, high priority traffic dominates low-priority traffic, thus, affecting the overall network throughput.

To address the problems posed by the IEEE 802.15.4 and PA-MAC , we propose a traffic-adaptive priority-based MAC protocol (TAP-MAC), which reduces the contention during the CAP period. The proposed MAC protocol provides a fair chance to the low-priority traffic by providing it with a dedicated contention access period during CAP. The new MAC protocol, called TAP-MAC, allocates CAP period to the low-priority traffic, according to the last estimated traffic load from low-priority and high priority traffic. We evaluate our proposed MAC by modifying and simulating the existing IEEE 802.15.4 MAC to prioritize WBAN traffic. Our simulation results show that the proposed TAP-MAC outperforms PA-MAC and IEEE 802.15.4 in terms of throughput, latency, and energy consumption in different scenarios.

The rest of the paper is organized as follows: Section 2 reviews the related works of priority-based MAC protocols. Section 3 presents the principles, design, and the detailed operation of the proposed traffic adaptive priority-based MAC protocol (TAP-MAC). Section 4 presents the performance analysis of TAP-MAC with the help of ns-3 simulations, and compares it with IEEE 802.15.4 [1] and the most recent priority-based MAC scheme, PA-MAC [8]. Finally, Section 5 concludes the paper and presents some future works.

## 2. Priority-Based MAC Protocols

Efficient design of priority-based MAC protocols for WBANs is a key challenge due to various application requirements including minimum energy consumption, minimum transmission delay, variable data rates, and critical data prioritization. In order to fulfill these requirements, several priority-based MAC protocols have been proposed in literature.

In [8], a priority-based WBAN MAC protocol called PA-MAC based on the IEEE 802.15.4 superframe has been proposed. PA-MAC divides the superframe into two access phases: CAP, and contention-free period (CFP). PA-MAC uses a dedicated control channel, and multiple data channels. Further, the CAP period is divided into four sub-phases according to the priority of the traffic. Traffic is classified into four different priorities; emergency traffic with priority one ($P_{1}$); on-demand traffic with priority two ($P_{2}$); normal traffic with priority three ($P_{3}$); and non-medical traffic with priority four ($P_{4}$). According to PA-MAC, traffic with priority $P_{1}$ can access all the four phases; traffic with priority $P_{2}$ can access from phase 2 to phase 4; and traffic with priority $P_{3}$ can access the channel during phase 3 and phase 4 only. Traffic with priority $P_{4}$ is given access to phase 4 only. PA-MAC supports high traffic very well, however, it pays little attention to low-priority traffic by restricting its access to phase 4 only, which affects the overall network throughput and latency. In majority of the WBAN scenarios, non-medical traffic constitutes the most of the traffic, and restricting this traffic to phase 4 may result in high contention, affecting the overall network latency, energy, and throughput.

In [9], S. Ullah et al. proposed a hybrid and a secure priority-based MAC protocol, called PMAC for WBANs. PMAC uses two CAP phases to facilitate life-critical and normal traffic, and one CFP to facilitate large data packets. The authors of [10] present a priority-guaranteed MAC protocol, in which data and control channels are separated to avoid collisions for higher data rate transmissions. The proposed protocol uses two data channels in CFP to handle two different classes of traffic: bursty and periodic traffic. Access to the CAP period is further distributed among two control channels: AC1 and AC2. In the proposed protocol, classification of traffic is based on periodicity or frequency of the traffic, and not on any quality of service (QoS) metrics. The protocol is based on IEEE 802.15.6 superframe, and to achieve reliable communication it uses priority control, length allocation, and time slot allocation dynamically for various access phases. As compared to the [9] based on EEE 802.15.4, in the IEEE 802.15.6 standard, a node’s priority depends on the type of information, and the node does not take into account variable data rate. Unlike [10], in another proposed work [11], both data rate and type of data are taken into consideration, while assigning priorities to different traffic classes. Detailed surveys on MAC protocol design and considerations for WBAN are presented in [12,13]. The survey work classifies and compares the existing protocols developed for WBAN, with a focus on their performance in WBAN under different scenarios.

Fang and Dutkiewicz in [14] present an energy-efficient time division multiple access (TDMA) based MAC protocol for WBAN. As compared to the [11], MAC superframe is divided into uplink and downlink sub-frames. To avoid idle listening, a sleep mode is also introduced in the superframe. Furthermore, in order to reduce packet collisions and control packet overhead, three bandwidth management schemes (adjusted bandwidth, burst bandwidth, and periodic bandwidth) have been used. A priority-based cross layer protocol called priority cross layer medium access channel protocol (PCLMAC) is presented in [15], where guaranteed efficient medium access and link formation is provided, while taking into consideration energy conservation, scheduling issues, and traffic types. PCLMAC tries to overcome idle listening and other limitations of the IEEE 802.15.6 and IEEE 802.15.4 standard. A priority-based protocol is presented in [16] that modifies the superframe structure of IEEE 802.15.4. Traffic is classified into different traffic categories according to the data rates of different nodes. Different nodes are assigned time slots from the superframe according to the priority. Unlike traffic priortization of [16], authors of [17], proposed a traffic load-aware sensor MAC (ATLAS) for WBANs. In ATLAS, the structure of superframe is adapted according to the traffic load to support multi-hop communication. Further, unlike [11,14,15], the ATLAS protocol also considers a load estimation and differentiation method to take into account the current network status.

In [18], a duty cycle learning algorithm (DCLA) is presented, which adapts duty cycle according to the last known activity. A key advantage of this algorithm compared to those found in [11,14,15,16] is that it does not need any external human intervention to activate the duty cycle. DCLA minimizes power consumption, and achieves high delivery ratio, and is suitable for wireless sensor platforms due to its lower memory and processing requirements. The author Xuedong Liang [19], investigates the practice of the IEEE 802.15.4 standard in ECG sensors in order to analyze the effect of carrier sense multiple access with collision avoidance (CSMA/CA) in terms of latency, delivery ratio, and energy efficiency. Unlike the work proposed in [19], the algorithm in [20] uses the CSMA/CA mechanism with service differentiation, and adaptive carrier sensing. The algorithm assigns different precedence levels to different sensor nodes according to specific QoS requirements. The proposed algorithm is based on two major phases; the first phase is based on service differentiation mechanism with diverse MAC parameters which are set according to different QoS requirements; and the second phase adjusts backoff according to different traffic conditions. In [21], Javier Espina et al. evaluate a WBAN network based on the IEEE 802.15.4 standard. The authors evaluate the performance of WBANs to monitor hypertension, cardiovascular, and stress detection. The extended contention cccess period (ECAP) algorithm [22] based on the IEEE 802.15.4 standard, targets bursty traffic in beacon-enabled mode, with a particular consideration to beacon order and superframe order. ECAP adjusts the active phase of a node according to the on-demand traffic, to support low latency real-time packet delivery. In [23], a protocol called H-MAC based on time division multiple access is presented. Compared to the ECAP, H-MAC targets to improve energy efficiency by allocating time slots, according to heartbeat pulse information. Results conducted by the authors reveal that peak pulses can be well harmonized with the time slots.

In [24], authors present a superframe duration adjustment scheme (SUDAS), with a specific focus on guaranteed time slot allocation, in a clustered tree network. The active part of the superframe is divided into three periods: CFP, CAP, and the beacon period (BP). Each node accesses the CAP period by using the CSMA/CA mechanism. Coordinator synchronizes the sensor nodes by broadcasting periodic beacon frames. A node may request for a guaranteed time slot for contention free transmission. According to the evaluation results of SUDAS, the longer CAP period is effectively utilized compared to IEEE 802.15.4 standard. The authors in [25], present a low delay traffic-adaptive media access control MAC protocol (LDTA-MAC) for WBAN. Unlike the IEEE 802.15.4 standard, the LDT-MAC superframe is based on dynamic Guaranteed Time Slot(GTS)allocation with fixed CAP duration. The active duration of superframe is adjusted based on traffic conditions. Authors in [26], proposed a novel channel access algorithm, which divides the CAP period in four phases on the basis of packet priorities, assigned by a WBAN coordnator. WBAN assigns 8 priorities to various packets which are: best effort, voice, high priority, excellent effort, network control, and video. According to [26], traffic is classified in four different levels varying from level 0 to level 4, depending on the priority of packets. During first phase of CAP; only level 0 traffic accesses the CAP; the second phase is accessed by level 0 and level 1 traffic; level 0 to level 2 traffic contend during the third phase; and all levels, i.e., level 0 to level 3 contend during phase 4 of CAP period. Results reveal that the proposed algorithm reduces power consumption under various scenarios. In [27], authors present an energy-efficient MAC protocol called 2L-MAC, which is based on two layers. The first layer deals with intra-WBAN based on a polling technique to coordinate data transfer, whereas the second layer is used by the coordinator to send polling frame to sensor nodes. The polling technique in 2L-MAC reduces the interference among nodes. The authors of [28], offer a dynamic beacon interval and superframe adaptation procedure (DBSAA), which adjusts duty cycle of nodes according to beacon order (BO) and superframe order (SO). In DBSAA, the coordinator considers packet collisions and number of packets successfully received to compute the duration of the next superframe. In [29], authors present an IEEE 802.15.4-based non-invasive scheme to continuously monitor and assess the blood pressure of patients.

From the above discussed literature, it is clear that majority of the priority-based MAC schemes based on IEEE 802.15.4, adjust the superframe according to traffic classifications, variable traffic loads, and traffic prioritization. However, we observe that most of these priority-based modifications to the IEEE 802.15.4 superframe structure only take care of highest priority traffic, while paying minimum or no attention to the low- priority traffic. Low-priority traffic is usually the highest load traffic in WBANs. Ignoring low-priority traffic altogether, while designing the IEEE 802.15.4 superframe may severely impair the overall network delay, energy consumption, and network throughput. We need to reconsider traffic-adaptive IEEE 802.15.4 superframe structure, which takes fairly good care of low- priority traffic, and allocates time slots fairly based on the low-priority traffic load. This fair slot allocation can create a balance between throughput and energy consumption, with a minimum possible delay under variable traffic loads.

## 3. IEEE 802.15.4 MAC and PA-MAC Overview

### 3.1. IEEE 802.15.4 MAC

IEEE 802.15.4 standard, a solution for low power, low rate wireless devices published in 2006, specifies the MAC and PHY for short range communication of 10 m [30]. Devices in IEEE 802.15.4 are classified as fully functional devices (FFDs) and reduced functional devices (RFDs). FFDs are capable to communicate with other devices as well to act as coordinator with full functionality, whereas RFDs can only be used as end devices. Communication between the coordinator and the end devices is possible through a superframe structure.

**Figure 1:**
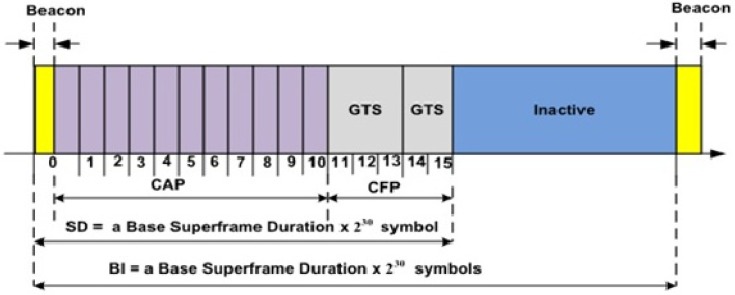
IEEE 802.15.4 superframe structure [1]. CAP: contention access period; CFP: contention-free period; GTS: gauranteed time slot; BI: beacon interval; SD: superframe duration.

The active section of the IEEE 802.15.4 MAC superframe structure consists of a beacon and two major access periods: CAP and CFP [31]. During, the CAP period, nodes contend the channel by using CSMA/CA, while during the CFP, nodes have a contention-free guaranteed channel access to deliver the data. The CFP period is mainly used for real-time or life-critical applications. The WBAN coordinator allocates up to seven guaranteed time slots as shown in Figure 1. Prior to the CFP period, the contention-based communication is completed, and each device willing to transmit in CAP makes sure that ongoing transmission is completed before the upcoming GTS slot [32]. The superframe structure of IEEE 802.15.4 in Figure 1 illustrates the active and inactive section of the superframe. Nodes transmit their data during the active part of the superframe, and if they find the carrier idle, they may switch to the sleep state during the inactive part of the superframe to conserve energy.

The IEEE 802.15.4 superframe structure depends tightly on two parameters: the SO and BO. The value of the SO and BO varies between 0 and 14, given that the BO is always greater than the SO. The beacon interval (BI) denoted as $T_{BI}$ can be calculated by using $T_{BI}=960\times 2^{BO}$ [33]. A WBAN coordinator maintains a list of nodes having a request to transmit important data on the first-come-first-serve basis, depends on the availability of the GTS slots [34]. If the coordinator notices that no GTS slots are available in the current superframe, it logs the received GTS request in the queue, and may allocate GTS slots to these requests in the upcoming superframe. If the number of requests exceeds buffer size, the next upcoming requests are dropped due to buffer overflow [35]. IEEE 802.15.4 MAC may operate in two modes:

1. Non beacon-enabled mode based on un-slotted CSMA/CA
2. Beacon-enabled mode, where sensor nodes receive periodic beacon frames from the coordinator to synchronize with the coordinator.

The IEEE 802.15.4-based WBAN utilizes binary backoff exponent during CSMA. Sensor nodes wait for a random backoff time to access the channel [36]. The backoff slot is set to 20 symbols, i.e., 320 μs, and with each subsequent collision this backoff duration is doubled [37]. For the uplink transmission, nodes contend the CAP by using the CSMA mechanism. However, for the downlink transmission, end nodes wait for an acknowledgement from the coordinator. Followed by the successful channel access either in the CAP or CFP period, the sensor node or coordinator transmits data according to the following data transfer models:

1. Whenever a node has data to transmit, the transfer is initiated by the coordinator.
2. If a coordinator has data to transmit, the transfer is still initiated by the coordinator.

In star topology, data transfer takes place between a node and the coordinator only. On the other hand, in tree-based topology in beacon-enabled mode, data is transferred to parents by descendant nodes who keep the footprint of beacon of the parent, and communicate with the coordinator announced in the previous beacon. In WBAN star topology, whenever a node wants to transmit data, it first sends request to the coordinator after listening the beacon message. The coordinator sends back an acknowledgement message to the corresponding node. Sensor nodes listen to the beacon structure, and wait for the data from a WBAN coordinator by sending a MAC appeal to avoid collisions. The coordinator sends an acknowledgement frame in response [38]. Afterwards, the coordinator sends any pending data to the sensor node. If data is successfully received by the node, it immediately sends an acknowledgment message to the coordinator.

### 3.2. PA-MAC

**Figure 2:**
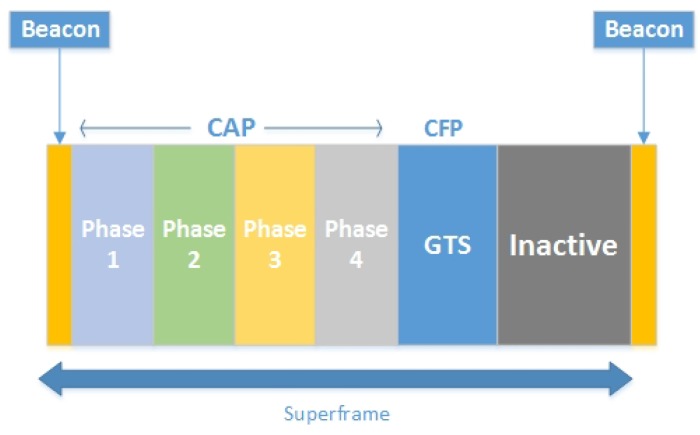
PA-MAC: priority adaptive MAC superframe structure [8].

In [8], authors propose a priority-based WBAN MAC protocol derived from the IEEE 802.15.4 superframe structure. Similar to the IEEE 802.15.4 superframe structure, the PA-MAC data channel also consists of two access periods, i.e., the CAP and CFP. Unlike the IEEE 802.15.4, the CAP period in PA-MAC is further subdivided into four sub-phases. Figure 2 illustrates the superframe structure of PA-MAC. PA-MAC prioritizes the traffic into four priority levels: emergency traffic with highest priority ($P_{1}$), on-demand traffic with priority ($P_{2}$), normal traffic with priority ($P_{3}$), and non-medical traffic with the lowest priority ($P_{4}$). Priority of nodes is set by using the association request command. Traffic with priority $P_{1}$ can access the CAP period during all the four phases; traffic with priority $P_{2}$ can access the CAP during phase 2 to phase 4; traffic with $P_{3}$ can access the CAP during phase 3 and phase 4 only; and traffic with priority $P_{4}$ is allowed to access during the phase 4 of CAP only.

Unlike IEEE 802.15.4 superframe, which does not take into account any priority of nodes, PA-MAC differentiates traffic according to the priority. However, in PA-MAC, the highest priority traffic is allowed to access during all the four phases of CAP, while low-priority traffic is restricted to the phase 4 only. This unfair CAP access based on traffic priorities may affect the low-priority traffic adversely, thereby degrading the overall throughput of the network.

## 4. Traffic-Adaptive Priority-Based MAC (TAP-MAC) Design and Operation

Unlike PA-MAC [8], our proposed protocol called TAP-MAC operates in partially connected mesh topology in the beacon-enabled mode. In our proposed scheme, the active period of the superframe consists of CAP and CFP. Furthermore, CAP is dynamically divided into CAP1 and CAP2 according to the traffic load observed by different traffic priorities. The detailed design and operation of TAP-MAC is discussed below.

### 4.1. TAP-MAC Design and Operation

Our proposed MAC protocol is based on traffic prioritization and traffic load to dynamically adjust the superframe structure in order to provide a fair chance to low-priority traffic, while still accommodating the emergency traffic.

#### 4.1.1. Traffic Classification

**Figure 3:**
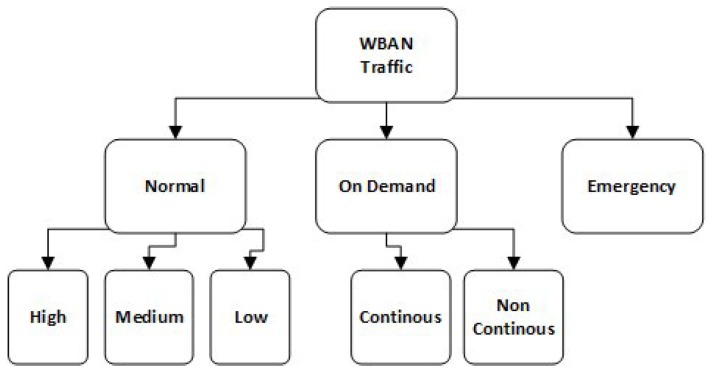
Traffic-adaptive priority-based MAC (TAP-MAC) traffic classification. WBAN: wireless body area network.

In our proposed TAP-MAC protocol, traffic is classified into three major classes: class 1 for emergency traffic, class 2 for on-demand traffic, and class 3 for normal traffic. On-demand traffic is further categorized as continuous and non-continuous. Further, normal traffic is categorized as low, medium, and high priority to fulfill the requirements of different applications of WBANs [39]. The classification used in TAP-MAC is illustrated in Figure 3.

#### 4.1.2. Traffic Prioritization

In TAP-MAC, traffic is prioritized into three priority levels: emergency traffic with the highest priority $P_{1}$, on-demand traffic with medium priority denoted as $P_{2}$, and normal traffic with lowest priority with $P_{3}$. Priority of nodes is decided at the application level by using special flags. The traffic priority levels used in TAP-MAC are briefly summarized in Table 1. Unlike PA-MAC [8], where traffic was classified into four traffic classes and four traffic priority levels, our proposed MAC is restricted to three priority classes.

#### 4.1.3. Dynamic CAP Adjustment

Unlike PA-MAC [8], in our proposed protocol, the CAP period is divided into two periods: CAP1 and CAP2. CAP1 deals with the emergency and on-demand traffic, whereas CAP2 deals with the normal traffic. CAP1 traffic is not allowed to access CAP2 period, and vice versa. The coordinator computes the superframe duration dynamically according to Algorithms 1 and 2. Initially, CAP is divided into CAP1 and CAP2 according to Algorithm 1. Further, whenever number of nodes in a WBAN vary, TAP-MAC calls Algorithm 1 to calculate the duration of CAP1 and CAP2, according to the traffic load and priority of nodes.

In Algorithm 1, CAP is divided into two different periods: $L_{CAP1}$ and $L_{CAP2}$, according to the number of nodes in each traffic category, i.e., $P_{1}$, $P_{2}$, and $P_{3}$ as listed in Table 1. Initially, if an association request is received from nodes with priority either $P_{1}$ or $P_{2}$, Algorithm 1 increments $N_{l}$. On the other hand, if an association request is received from the nodes with priority $P_{3}$, Algorithm 1 increments $N_{k}$. The coordinator computes the length of CAP1 and CAP2, according to the ratio of number of nodes in $N_{l}$ and $N_{k}$, to the total number of nodes $N_{T}$ in the WBAN. At the end, Algorithm 1 calls Algorithm 2 to compute the lengths of sub-phases of each traffic category.
**Algorithm 1:** Computes $L_{CAP1}$ and $L_{CAP2}$ based on traffic conditions. 
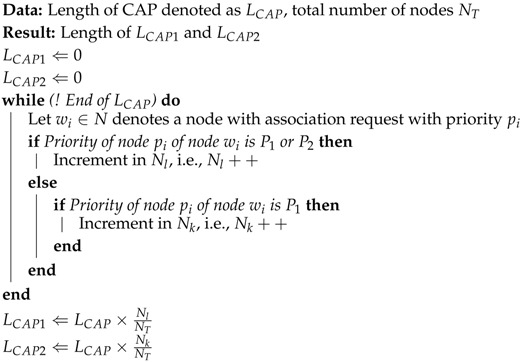



In Algorithm 2, the CAP1 period is further sub-divided into two sub-phases: phase 1 for priority $P_{1}$ nodes, and phase 2 for priority $P_{2}$ nodes. For the first superframe, CAP1 and CAP2 are calculated according to Algorithm 1. The coordinator takes the request from the nodes with the priority $P_{1}$ and $P_{2}$ during $L_{CAP1}$, and calculates the number of sub-phases denoted as $l_{P_{i}}$ from the $L_{CAP1}$, according to the number of requests received from the either priority class, i.e., $P_{1}$ or $P_{2}$. Further, assuming that only $P_{3}$ nodes access the CAP2 period, Algorithm 2 also calculates the sub-phases for the $P_{3}$ nodes from the CAP2. At the end, Algorithm 2 also updates the length of $L_{CAP1}$ and $L_{CAP2}$ without calling Algorithm 1, therefore avoiding unnecessary delays by using the most recent traffic load from each of the traffic classes.
**Algorithm 2:** Computes sub-phases $l_{P_{i}}$ of $L_{CAP1}$ and $L_{CAP2}$ for i=1,2,3. 
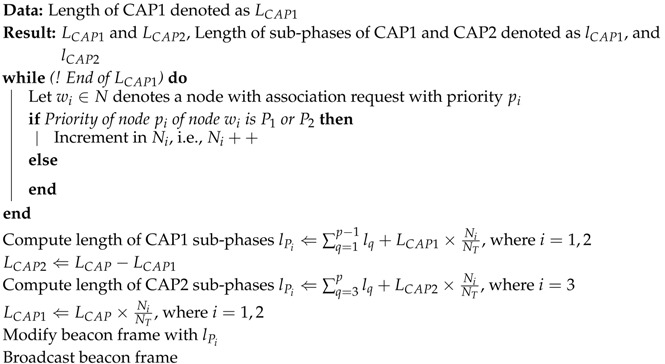



#### 4.1.4. TAP-MAC Superframe Structure

**Figure 4:**
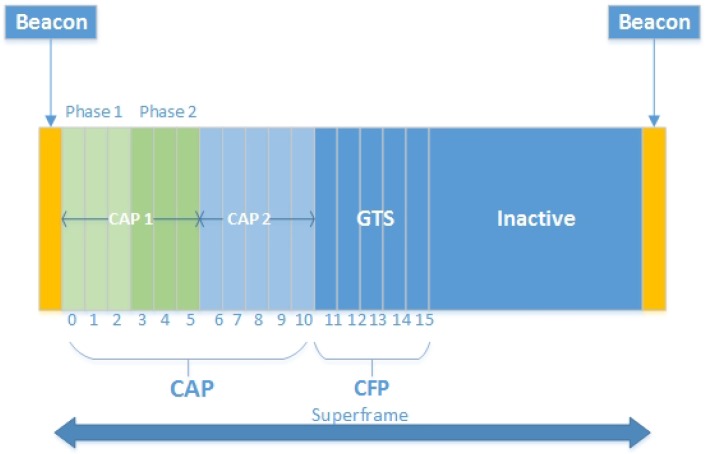
The TAP-MAC superframe structure. CAP1: contention access period 1; CAP2: contention access period 2.

Similar to IEEE 802.15.4 beacon-enabled mode, the TAP-MAC superframe starts with a beacon which carries synchronization information. However, unlike IEEE 802.15.4, in the proposed protocol, CAP is further sub-divided into two periods: CAP1 and CAP2, for different levels of traffic by using Algorithm 1 for the first superframe, and Algorithm 2 for the subsequent superframes. From the 16 slots of the superframe, 5 GTS slots are used for contention free communication, while remaining 11 time slots are used for the CAP. These 11 slots are further sub-divided into CAP1 and CAP2 dynamically by using Algorithms 1 and 2, where CAP1 is used by $P_{1}$ and $P_{2}$ traffic, and CAP2 is exclusively used by the $P_{3}$ traffic. Further, similar to the IEEE 802.15.4, in TAP-MAC the CFP period is followed by an inactive period. During an inactive period, nodes turn their radio off to conserve energy. The modified superframe structure of TAP-MAC is shown in Figure 4.

#### 4.1.5. Channels in TAP-MAC

**Figure 5:**
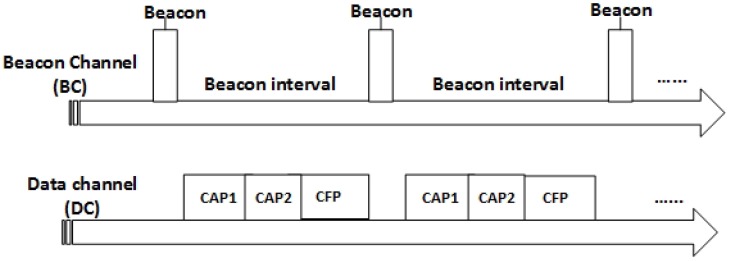
Channels in TAP-MAC.

Similar to the IEEE 802.15.4 radio hardware, TAP-MAC uses a beacon channel (BC) and a data channel (DC). However, unlike IEEE 802.15.4, the data channel in TAP-MAC is modified according to the modified TAP-MAC superframe structure as discussed earlier. In TAP-MAC, a beacon channel is used to broadcast beacon frames and other control information in the network, whereas a data channel is used to transfer data. At the beginning of the beacon period, nodes switch to the specified beacon channel to synchronize with the coordinator. Nodes switch to their respective data channels based on the synchronization information received through the beacons to receive or send data. Nodes can switch between the data channel and beacon channels by using a negligible switching delay, as illustrated in Figure 5. A beacon channel scan can provide the information about the whole network. In order to minimize the interference caused by wireless Lan (WLAN), TAP-MAC uses channel 25 and channel 26, coupled with the low transmission power for the beacon channel.

#### 4.1.6. TAP-MAC Data Transfer

**Figure 6:**
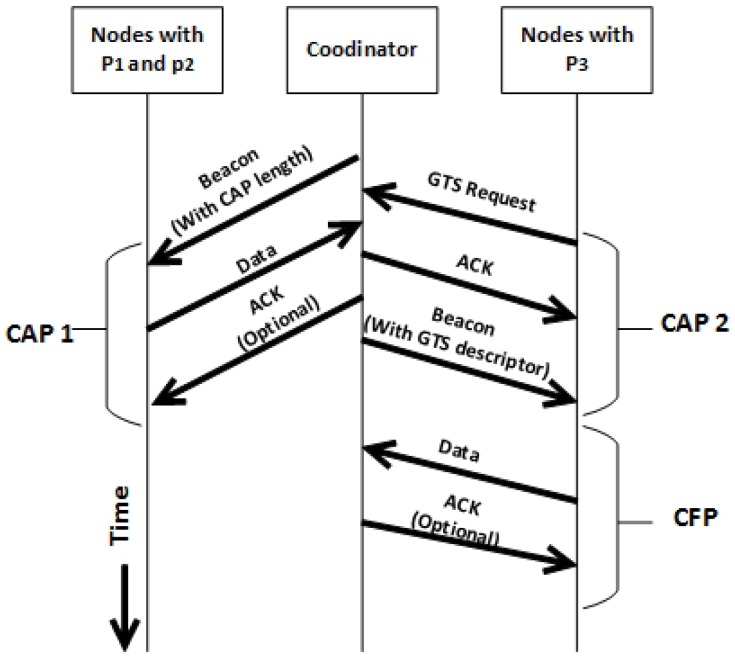
TAP-MAC data transfer. ACK: acknowledgment.

In TAP-MAC superframe structure, three access periods available for the data transmission of data are: CAP1, CAP2, and CFP. In TAP-MAC, CAP1 and CAP2 are mainly used to send small data packets; however for longer, continuous, and real-time traffic, CFP is preferred. During CAP1 and CAP2, all nodes with different traffic priorities access the channel by using the CSMA/CA procedure. However, during the CFP period, nodes transfer data according to the GTS slots allocated by the coordinator, and therefore are guaranteed to transfer collision-free. In order to send GTS slot allocation request to the coordinator, nodes contend for the channel by using the CSMA/CA during CAP1 and CAP2 periods according to node priorities. Figure 6 shows communication between nodes and the coordinator. Figure 6 illustrates that nodes with priorities $P_{1}$ and $P_{2}$ access the channel during CAP1, and therefore send data during the CAP1 period. However, data from $P_{3}$ nodes is deferred until the CAP2 period, which is exclusively used to handle the data transfer and GTS requests from the priority $P_{3}$ nodes.

## 5. TAP-MAC Performance Evaluation

The TAP-MAC protocol can be implemented by using the popular transceivers, used for the short range and low-power WPANs, including CC2420 and CC2500 [40].

In this section, we present simulations of proposed MAC protocol TAP-MAC, and compare it with the IEEE 802.15.4 [41] and PA-MAC [8]. All our simulation results are averaged with a confidence level of 95% by running each simulation scenario 10 times. We use three performance metrics: network throughput, average end-to-end delay, and average energy consumption, for the evaluation of all three MAC protocols.

### 5.1. Simulation Environment

We evaluate the performance of TAP-MAC, and compare it with other MAC protocols by using a simulator, ns-3.20. ns-3 is an open source simulator and is based on ns-2 with the aim to replace ns-2. ns-3 supports simulation of wireless networks including low rate low power personal area networks. The core of ns-3 is implemented by using C++ and python scripting interface.

The majority of WBAN MAC protocols including PA-MAC are evaluated under star topology. However, we consider partially connected mesh topology for the evaluations of all the three schemes in the beacon-enabled mode. It is assumed that WBAN coordinator has knowledge of traffic type, data rate, and response time of each node. It is also assumed that all the nodes are in the range of WBAN coordinator with no effect on their communication due to mobility or gesture change, and operate in 2.4 GHz-band, with a data rate of 250 kbps. Further, nodes in our network are assumed to generate only one type of traffic. Moreover, traffic in our simulated network is generated under ideal conditions, without any interference from any other network.

**Figure 7:**
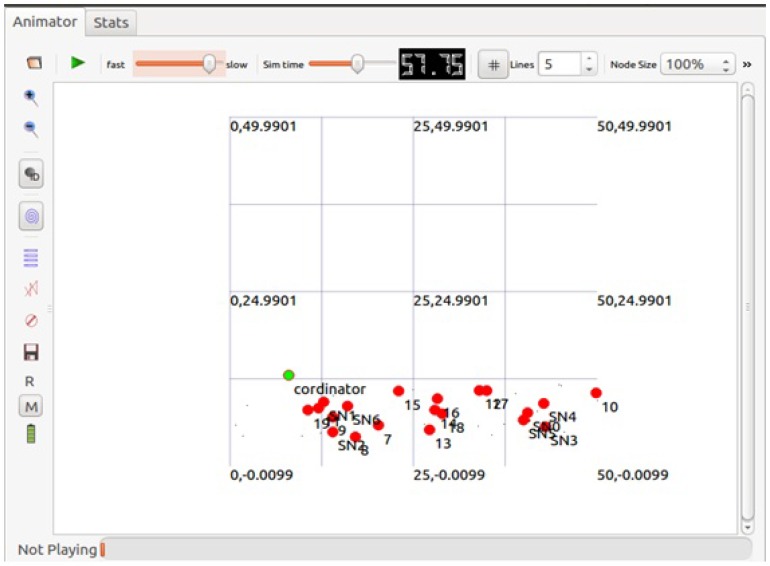
ns-3.20 nam window.

We assume that all the bio-medical sensors are attached to a human body. Mesh topology, used in our experiments, consists of a WBAN coordinator indicated with the green color, and remaining sensor nodes are randomly placed around the coordinator, within an area of 4 m radius as shown in Figure 7. Each simulation runs for 100 s, with an initial energy of 1 Joule, where energy consumption of each node is modeled by using the ATMEL energy model, listed in Table 2 [42].

The detailed simulation parameters for the performance evaluation of TAP-MAC ,and other MAC protocols are listed in Table 2. Packet size for all the scenarios is 512 bytes, if not stated otherwise. The active period of the superframe consists of 16 time slots, each consisting of 20 symbol periods individually, called a unit backoff period. In 2.4-GHz operated IEEE 802.15.4, each symbol consists of 4 bits of data, with a symbol rate of 62.5 (ksymbols/s) [43].

### 5.2. Simulation Scenarios

We evaluate the performance of proposed MAC under different scenarios of varying number of nodes and packet size. For all the three scenarios, we select the maximum number of nodes as 60 because under 60, TAP-MAC and other competitive MAC protocols, i.e., PA-MAC and IEEE 802.15.4 show performance variations. However, we observe that for all these scenarios, when the number of nodes exceed 60, average network throughput degrades, with higher delay and high energy consumption, as more nodes contend for the CAP access. These three scenarios are discussed below:

#### 5.2.1. Scenario 1

In our first scenario, TAP-MAC protocol, IEEE 802.15.4 [1], and priority-based, PA-MAC [8] are simulated, under constant packet size of 256 bytes, with varying number of nodes from 10,20,30,40,50, and 60. Other simulation parameters for all three MAC protocols are same as those listed in Table 2.

##### Average Network Throughput

**Figure 8:**
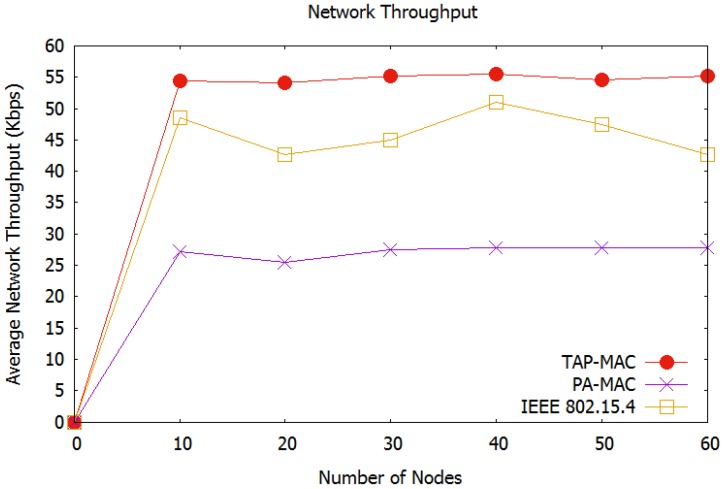
Scenario 1: Average network throughput.

Figure 8, illustrates the average network throughput observed in our first scenario. In TAP-MAC, access to the CAP is restricted according to the priority of the nodes. Further, access to CAP1 is restricted to $P_{1}$ and $P_{2}$ traffic, and access to the CAP2 is restricted to $P_{3}$ traffic. On one hand, this restricted access reduces the number of collisions experienced by a WBAN, and on the other hand it provides a fair chance to $P_{3}$ traffic, thereby improving the average network throughput. The proposed TAP-MAC performs better compared to the IEEE 802.15.4 MAC, which provides no fair channel access to the low-priority traffic, and therefore, experiences a higher number of collisions. TAP-MAC also observes better throughput, compared to the priority-based PA-MAC, which also does not provide a fair chance to low-priority traffic. We observe that even a priority-based MAC protocol, e.g., PA-MAC, may result in higher number of collisions, if the access to the CAP is not well managed for low-priority traffic. Figure 8, illustrates that for 30 nodes, TAP-MAC achieves 30% higher throughput compared to the PA-MAC. Additionally, the average network throughput exhibited by TAP-MAC stabilizes with an increase in the number of nodes.

##### Average Network Delay

**Figure 9:**
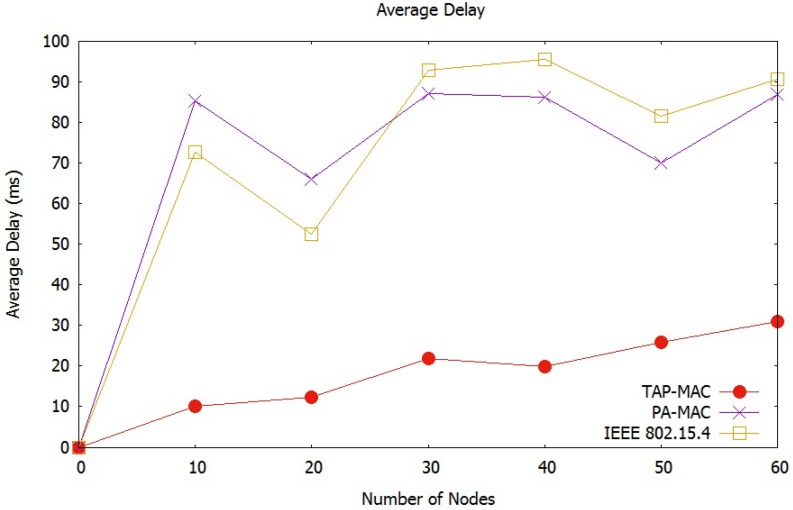
Scenario 1: Average network delay.

The average network delay observed by the scenario 1 is illustrated in Figure 9. Figure 9 shows an increase in average network delay, with an increase in the number of nodes. An increase in the number of nodes increases contention to the CAP, resulting in a higher number of collisions, and therefore higher delay. In the proposed TAP-MAC, contention to the CAP is significantly less than IEEE 802.15.4 MAC and PA-MAC, due to restricted access of traffic during CAP1 and CAP2. In PA-MAC, CAP sub-phases experience higher contention compared to TAP-MAC, due to unrestricted access of different traffic classes to the CAP, resulting in a higher number of collisions. Figure 9 shows that the average network delay experienced by IEEE 802.15.4 MAC is the highest among all three protocols, as the number of nodes increases. IEEE 802.15.4 MAC operates without any priority-based channel access, and therefore exhibits a higher delay. We observe from Figure 9 that for 40 nodes PA-MAC and IEEE 802.15.4 experience a delay of almost 85 ms and 95 ms, respectively; while TAP-MAC outperforms with an average delay of 20 ms by providing a fair chance to low-priority traffic during CAP2.

##### Average Energy Consumption

**Figure 10:**
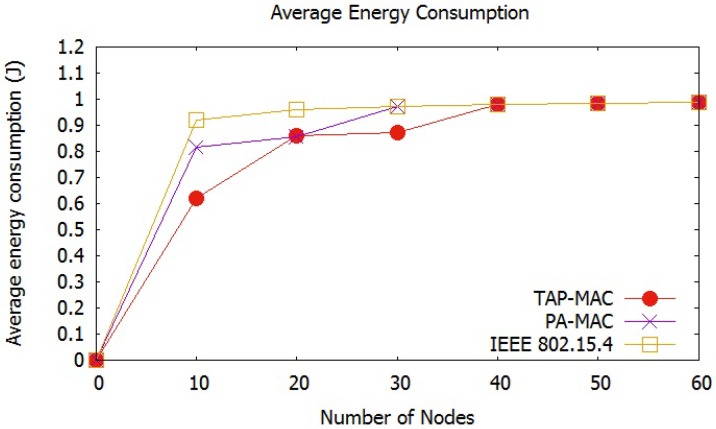
Scenario 1: Average energy consumption.

Energy consumption of a sensor node depends on the energy consumed during transmitting, receiving, idle, and sleep states [42]. Energy consumption for the evaluation of all the three MAC protocols is calculated using ATMEL energy model, listed in Table 2. Figure 10, shows that the average energy consumption experienced by all the three MAC protocols increases with an increase in the number of nodes. An increase in the number of nodes in the network may increase the traffic, which increases the number of collisions, and therefore, the number of re-transmissions. Figure 10, shows that the IEEE 802.15.4 MAC protocol experiences highest energy consumption with an increase in the number of nodes. For 20 nodes, IEEE 802.15.4 consumes almost 1 joule of energy, while for the same case PA-MAC and TAP-MAC consumes less than 0.8 joules of energy. The dynamic CAP adjustment, and the restricted access of TAP-MAC to CAP, significantly reduces the contention level, which results in a lower number of collisions, thereby resulting in fewer re-transmissions. The proposed TAP-MAC and PA-MAC, owing to its fair chance to low-priority traffic during CAP2, results in less energy consumption compared to the IEEE 802.15.4.

#### 5.2.2. Scenario 2

In our second scenario, the TAP-MAC protocol is simulated under a constant packet size of 512 bytes, with a varying number of nodes from 10,20,30,40,50, and 60. The other simulation parameters are same as those shown in Table 2. The set of results for TAP-MAC are compared with IEEE 802.15.4 [1], and priority-based PA-MAC [8].

##### Average Network Throughput

**Figure 11:**
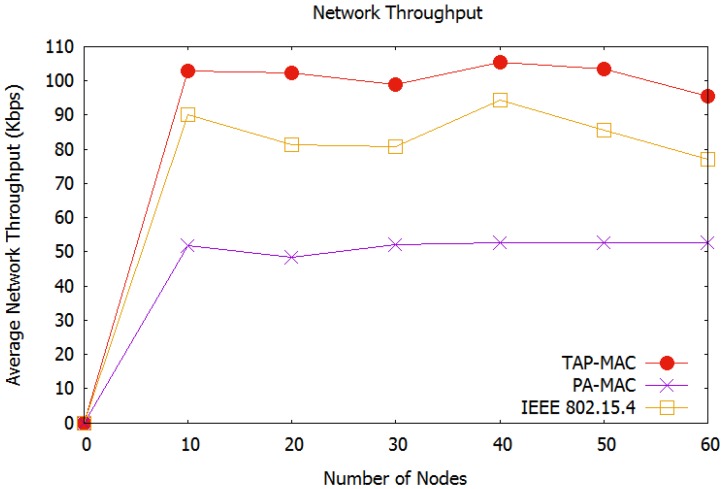
Scenario-2 average network throughput.

Average network throughput of the second simulated scenario is shown in Figure 11. All three MAC schemes show a similar average network throughput trend, with an increase in the number of nodes in the network. For all the three MAC schemes, there is an increase in network throughput when there are between 40 and 50 nodes. However, it degrades significantly when the number of nodes exceeds 50. When the number of nodes exceeds 50, more nodes contend for the channel access, which increases collision probability, thereby degrading the overall network throughput. The proposed TAP-MAC, and IEEE 802.15.4 MAC protocol, however, provide better throughput compared to the PA-MAC. The number of collisions increases with an increase in the number of nodes and packet size for all the three MAC protocols, however, the access to the data channels is well managed in TAP-MAC. This restricted access to CAP2 for low-priority traffic improves the overall network throughput.

##### Average Network Delay

**Figure 12:**
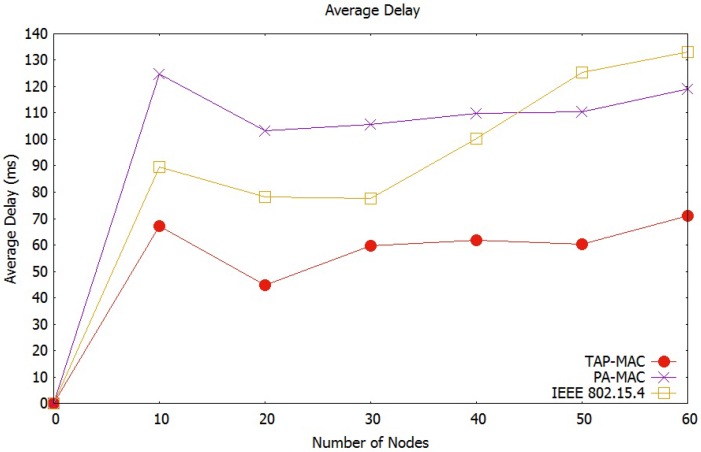
Scenario 2: Average network delay.

The average network delay, experienced by all the three MAC schemes for scenario 2, is shown in Figure 12. There is a direct relationship between the traffic generated by nodes, and the average network delay. The higher the number of nodes in the network, the higher the traffic generated, and therefore the higher the contention, resulting in higher delay experienced by all the three MAC protocols. If the channel contention in the network increases, the sensor nodes tend to back off for longer periods to compete for the channel access, resulting in longer access delays. Figure 12 shows that when the number of nodes is 30, IEEE 802.15.4 experiences 20%, and PA-MAC 30% higher delays compared to the TAP-MAC. Even for a larger packet size, i.e., 512 bytes, TAP-MAC achieves less of a delay compared to the IEEE 802.15.4 and PA-MAC due to minimum number of collisions, owing to its well managed channel access for low-priority traffic. IEEE 802.15.4 has a higher delay in this scenario as compared to our first scenario, i.e., when packet size changes from 256 bytes to 512 bytes.

##### Average Energy Consumption

**Figure 13:**
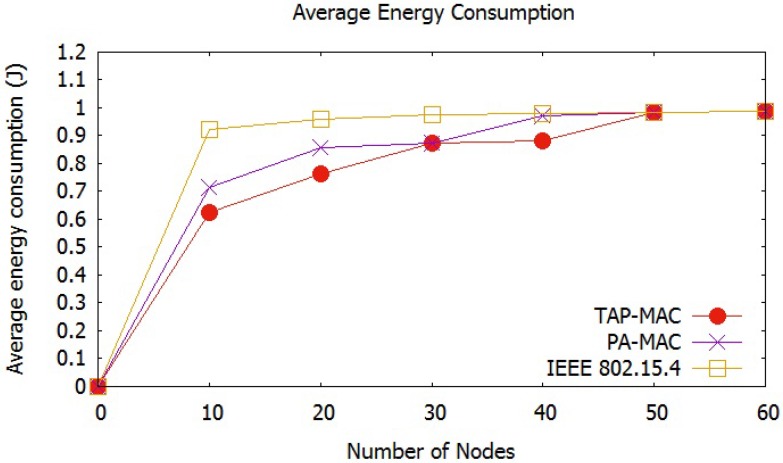
Scenario 2: Average energy consumption.

Figure 13 shows the average energy consumption with varying number of nodes for the TAP-MAC, IEEE 802.15.4, and priority-based PA-MAC. A higher collision ratio, higher number of re-transmissions, and larger packet size are the key contributors of higher energy consumption. The larger the packet size, the more processing is required by the sensor nodes, thereby resulting in higher energy consumption. In scenario 2, PA-MAC is more energy-efficient compared to the IEEE 802.15.4. For 30 nodes, PA-MAC and TAP-MAC show similar energy consumption, however for 40 nodes, PA-MAC consumes less than 0.9 joules of energy, while the energy consumption of TAP-MAC remains at 0.8 joules. The TAP-MAC successful data transmission rate is better, and there is less of a chance of re-transmissions, which results in lower energy consumption. However, similarly to other MAC protocols, TAP-MAC consumes 1 joules of energy when the number of nodes exceeds 50.

#### 5.2.3. Scenario 3

In our third and last scenario, TAP-MAC protocol is simulated under a constant packet size of 1024 bytes, with varying number of nodes from 10,20,30,40,50, and 60. Other simulation parameters are same as listed in Table 2. The set of results are compared with the IEEE 802.15.4 [1], and priority-based PA-MAC [8].

##### Average Network Throughput

**Figure 14:**
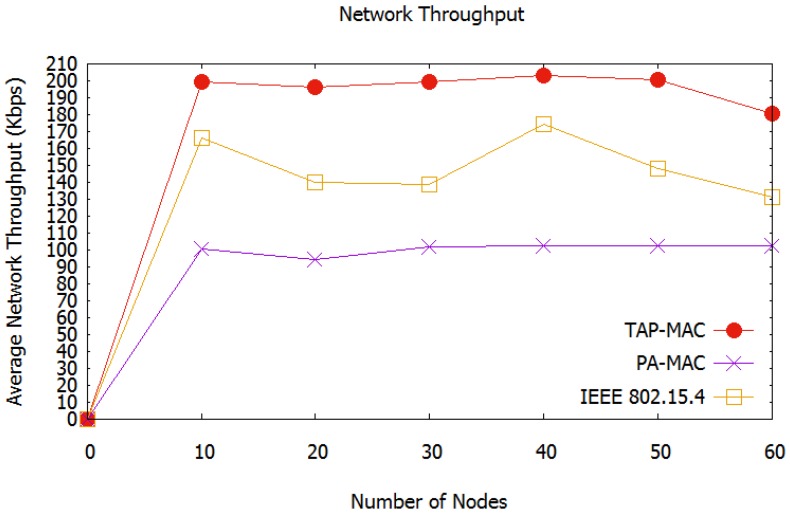
Scenario 3: Average network throughput.

The average network throughput of the third scenario is shown in Figure 14. In case of TAP-MAC, restricted access to CAP2 for low-priority traffic significantly reduces the number of collisions, which improves the average network throughput compared to PA-MAC and IEEE 802.15.4. The average network throughput achieved by PA-MAC is lower due to a higher number of collisions and unfair chance to low-priority traffic compared to the TAP-MAC. For 40 number of nodes, TAP-MAC achieves a throughput of 195 kbps, while PA-MAC shows a throughput of 95 kbps, and the IEEE 802.15.4 demonstrates a throughput of 170 kbps. TAP-MAC achieves overall better network throughput by providing a fair chance to $P_{3}$ traffic during the CAP2 period. Even for an increase in packet size from 256 to 512, and to 1024 bytes, TAP-MAC still achieves highest throughput from 10 to 50 nodes, compared to PA-MAC and IEEE 802.15.4.

##### Average Network Delay

**Figure 15:**
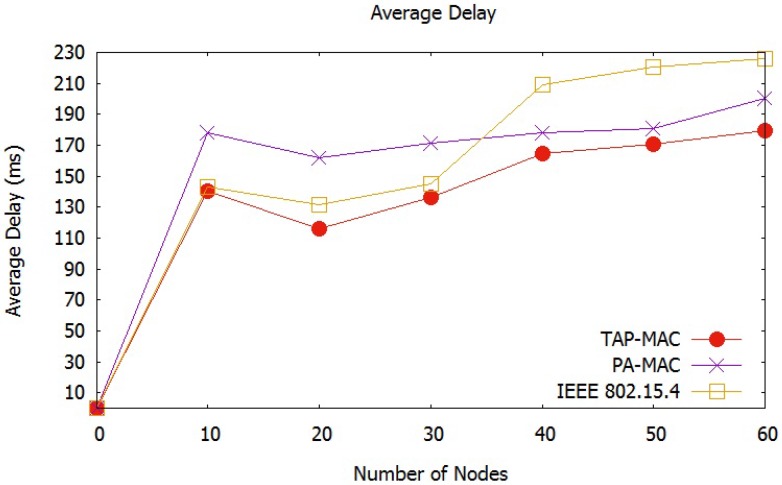
Scenario 3: Average network delay.

Average network delay observed by our third scenario is shown in Figure 15. Overall, the average network delay for all the three MAC schemes is high due to larger packet size of 1024 bytes used in this scenario, compared to scenario 1 and scenario 2, owing to extra processing for larger packet size in scenario 3. For 30 nodes, PA-MAC has the highest delay, and it increases with an increase in the number of nodes. However, IEEE 802.15.4 has less delay when the number of nodes are 30, however as the number of nodes approach 40, the delay experienced by IEEE 802.15.4 is highest, compared to PA-MAC and TAP-MAC. TAP-MAC demonstrates an initial delay of 130 ms when the number of nodes are 30, however it increases to 170 ms when the number of nodes exceeds 50. TAP-MAC performs better in larger networks compared to other two MAC protocols due to fewer collisions, which is mainly attributed to the fair chance to low-priority traffic during CAP2 access.

##### Average Energy Consumption

**Figure 16:**
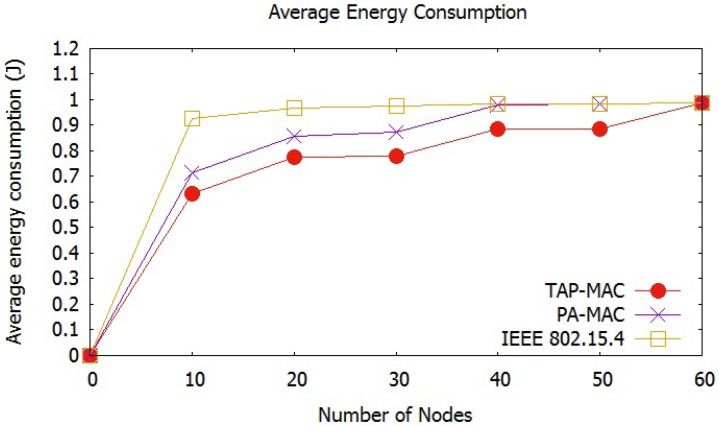
Scenario 3: Average energy consumption.

Average energy consumption of scenario 3 is illustrated in Figure 16. Energy consumption depends on the ratio of collisions, and therefore on the number of re-transmissions. Energy consumption is higher in networks with higher traffic, compared to for less traffic. In previous scenarios, i.e., scenario 1 and scenario 2, PA-MAC performs the worst, however it is energy efficient compared to the IEEE 802.15.4 due to the prioritization of traffic. When the number of nodes is 30, IEEE 802.15.4 consumes approximately 1 joule of energy, while PA-MAC consumes only 0.8 joules, and TAP-MAC consumes less than 0.7 joules of energy. TAP-MAC performs better in terms of energy compared to the IEEE 802.15.4 and PA-MAC due to its traffic prioritization, and more importantly fair channel access to low-priority traffic during CAP2. However, when the number of nodes exceeds 50, TAP-MAC tends to consume more energy, similar to IEEE 802.15.4 and PA-MAC.

## 6. Conclusions

Unlike IEEE 802.15.6, IEEE 802.15.4 does not offer traffic classification and prioritization, and therefore may not seem to fullfil the requirements of medical and healthcare applications. However, its unique PHY and MAC layer characteristics and its maturity has stimulated the use of the IEEE 802.15.4 standard for wireless body area networks. While IEEE 802.15.4 does not offer traffic prioritization directly, it can be well modified to accommodate the medical and healthcare application requirements. Recently, MAC protocols, mainly derived from the superframe structure of IEEE 802.15.4, have been introduced. These protocols offer distinguished QoS to various traffic types that may exist in a WBAN. One of the recent and popular modifications to the IEEE 802.15.4 superframe is PA-MAC, which divides the CAP in four sub-phases where traffic with highest priority is dominant over low- priority traffic and may adversely affect the overall network performance. In this paper, we propose a subtle modification to the IEEE 802.15.4 MAC that dynamically divides the CAP period into CAP1 and CAP2, according to the low-priority and high priority traffic load, and provides a very fair chance to low-priority traffic. In our modified MAC superframe structure, low-priority traffic which is the usually the largest volume traffic in the network, exclusively accesses the CAP2 period to transmit data. The well managed and dynamic CAP period in our proposed MAC offers highly reliable, energy-efficient, low-latency transmission. We carried out extensive simulation to evaluate the performance of the proposed modification called TAP-MAC. We also compared our proposed MAC protocol with the conventional IEEE 802.15.4, and a most recent priority-based PA-MAC. The simulation results indicate that TAP-MAC performs better in terms of average network throughput, energy efficiency, and end-to-end delay, compared to the IEEE 802.15.4 and PA-MAC under different scenarios of varying traffic load.

Similar to a most recent work on decentralized time-synchronized channel swapping (DT-SCS) for ad hoc wireless networks [44], it will be interesting to explore time-synchronized channel hopping with TAP-MAC. The DT-SCS mechanism can enable a node to spontaneously adapt to packet losses in a decentralized way, without any reliance on a coordinator. Further, it is possible to improve the energy efficiency of TAP-MAC by exploiting the Q-learning approach [45]. Q-learning-based TAP-MAC can reduce a node’s idle listening by exploiting cross-layer neighborhood traffic load information from the network layer, and adapting its wake-up strategy to it. To conclude, it is also possible to modify TAP-MAC to handle emergency traffic during the CAP2 period, with a minimal effect on the low-priority traffic.

## Figures and Tables

**Table 1:** TAP-MAC traffic priority levels. EMG: electromyogram; EEG:electroencephalogram.

Traffic	Priority Level	Examples
Emergency traffic	$P_{1}$ (Highest)	Emergency alarm signals
On-demand traffic	$P_{2}$ (Medium)	Continuous/non-continuous medical signals (EEG, EMG, blood pressure, temperature)
Normal traffic	$P_{3}$ (Lowest)	Audio/video/data

**Table 2:** Simulation parameters.

Parameter	Value
Data rate	250 Kbps
Frequency	2.4 GHz
Symbol rate	62.5 ksymbols/s
Superframe duration	122.88 ms
Transition time	192 μs
aUnitBackoff period	20 symbols
macMaxCSMABackoffs	5
macMinBE	3
macMaxBE	5
Initial energy	1 Joule
Transmission power consumption	12.3 mA
Reception power consumption	14 mA
Idle power consumption	0.4 mA
Traffic type	CBR
Clear channel assessment	8 symbols
Beacon size	40 bytes
